# Carotenoid distribution in wild Japanese tree frogs (*Hyla japonica*) exposed to ionizing radiation in Fukushima

**DOI:** 10.1038/s41598-018-25495-5

**Published:** 2018-05-09

**Authors:** Mathieu Giraudeau, Jean-Marc Bonzom, Simon Ducatez, Karine Beaugelin-Seiller, Pierre Deviche, Thierry Lengagne, Isabelle Cavalie, Virginie Camilleri, Christelle Adam-Guillermin, Kevin J. McGraw

**Affiliations:** 1 School of Life Sciences, Arizona State University, Tempe, AZ 85287-4501 USA; 2 Centre for Ecology & Conservation, College of Life and Environmental Sciences, University of Exeter, Penryn, UK; 3 Institut de Radioprotection et de Sûreté Nucléaire (IRSN), PSE-ENV/SRTE/LECO, Cadarache, 13115 Saint Paul Lez Durance, France; 4 School of Biological Sciences A08, University of Sydney, Sydney, NSW 2006 Australia; 5 Université de Lyon 1, CNRS, UMR 5023, Laboratoire d’Ecologie des Hydrosystèmes Naturels et Anthropisés, Bât. Darwin C, F-69622 Villeurbanne Cedex, France

## Abstract

The nuclear accident in the Fukushima prefecture released a large amount of artificial radionuclides that might have short- and long-term biological effects on wildlife. Ionizing radiation can be a harmful source of reactive oxygen species, and previous studies have already shown reduced fitness effects in exposed animals in Chernobyl. Due to their potential health benefits, carotenoid pigments might be used by animals to limit detrimental effects of ionizing radiation exposure. Here, we examined concentrations of carotenoids in blood (i.e. a snapshot of levels in circulation), liver (endogenous carotenoid reserves), and the vocal sac skin (sexual signal) in relation to the total radiation dose rates absorbed by individual (TDR from 0.2 to 34 µGy/h) Japanese tree frogs (*Hyla japonica*). We found high within-site variability of TDRs, but no significant effects of the TDR on tissue carotenoid levels, suggesting that carotenoid distribution in amphibians might be less sensitive to ionizing radiation exposure than in other organisms or that the potential deleterious effects of radiation exposure might be less significant or more difficult to detect in Fukushima than in Chernobyl due to, among other things, differences in the abundance and mixture of each radionuclide.

## Introduction

The nuclear accident at the Fukushima Dai-ichi nuclear power plant (NPP) on March 11, 2011 released large amounts of artificial radionuclides into the environment, polluting the Pacific Ocean and thousands of km^2^ of land in Japan^1–6^. The total activity of radionuclides released was estimated at about 520 (a range of 340–800) PBq^7^ and the size of the continental contamination zone with levels ≥185 kBq/m2 is defined by an area of approximately 1700 km^2^ (<75% of forest, ≤10% rice paddy fields, ≤10% other agricultural areas, and ≤5% urban areas)^8^. This contamination event provides a rare opportunity to study the ecological, physiological, and evolutionary consequences of ionizing radiation exposure on living organisms. The other major nuclear accident, which occurred in Chernobyl in 1986, has been extensively studied, but research on the effect of chronic radiation exposure on wild vertebrates was almost absent for the first ten years following the disaster (i.e., Chernobyl research on animals began in 1998^9^). Thus, the Fukushima prefecture accident constitutes an opportunity to understand and predict the short- and long-term biological effects of exposure to artificial radionuclides on wildlife.

To date, few studies have assessed the biological effects of the radionuclides released by the accident at the Fukushima Dai-ichi NPP on free-ranging organisms. There is evidence of a transient increased occurrence of morphological abnormalities in pale grass blue butterflies (*Pseudozizeeria maha*^10,11^, see also in gall-forming aphid (*Tetraneura sorin*)^12^), of lower white and red blood cell counts, hemoglobin and hematocrit levels in wild monkeys^13^, and a reduced abundance of birds and insects associated with an increased level of ambient dose rate^14–16^. Garnier-Laplace *et al*.^17^ also recently showed that the overall abundance of birds at Fukushima during 2011–2014 decreased with increasing absorbed doses. These studies show clear effects of the Fukushima Dai-ichi NPP disaster on several wild species, but the mechanistic basis for these effects remains unclear. Studies that probe how radiation exposure affects resource allocation between processes linked to self-maintenance or reproduction may provide deeper insights into the physiological and evolutionary responses of wild animals to environmental contamination.

Ionizing radiation can be a source of harmful reactive oxygen species (ROS) in living cells^18,19^, and several studies have now shown that animals living in the Chernobyl Exclusion Zone showed reduced levels of dietary antioxidants^20,21^, leading to elevated levels of oxidative stress that may explain the reduced fitness (e.g. survival and reproduction) and mutagenetic effects (DNA mutation) observed in these exposed individuals^22–25^. In line with these findings in Chernobyl, a first study on streaked shearwaters chicks (*Calonectris leucomelas*) from Mikura Island - located approximately 220 km south of Tokyo, and within the Fukushima nuclear plume -, four to seven months after the Fukushima nuclear accident, showed that they displayed significantly reduced vitamin A levels compared to the breeding colony on Birou Island, which lies outside the affected zone (Uematsu *et al*.^26^). However, only the ambient external dose was measured (0.026 vs 0.143 µGy/h, respectively, for the Birou Island control and Mikura Island exposed groups). Therefore, the estimate of absorbed dose is incomplete, since radionuclide contamination (for example, by ingestion of contaminated food) and subsequent internal dose rate have not been estimated.

Carotenoids are especially interesting in the context of the Fukushima nuclear disaster because they are responsible for many red, orange, and yellow colors widely used as a signal of sexual attractiveness^27^ and can have health benefits (though their antioxidant properties are still debated; see Tomášek *et al*.^28^, Costantini and Møller^29^). There are several non-mutually exclusive mechanisms by which radiation exposure may alter or modify carotenoid allocation in animals. First, dietary availability of carotenoid pigments may be reduced if ionizing radiation impacts the fitness (i.e. abundance) or carotenoid status (e.g. health state) of food sources (e.g. plants, insects). In line with this hypothesis, Møller and Mousseau^30^ showed that the abundance of potential invertebrate prey (e.g. bumblebees, butterflies, grasshoppers, dragonflies, and spiders) decreased with increasing ambient external dose rate around Chernobyl. However, the dietary availability of carotenoids contained in such prey within contaminated and control areas has never been compared in any studies at Chernobyl or Fukushima. Second, carotenoid pigments in the body (and those available for coloration) may be drained to limit the negative impact of an exposure to ionizing radiations on health. Consistent with this idea, ambient dose rate measured at ground level positively predicted plasma concentration of reactive oxygen metabolites in barn swallows (*Hirundo rustica*) in Chernobyl^21^. Alternatively, under the terminal investment hypothesis^31–33^, individuals in highly contaminated areas and with a reduced survival prospect may maximize their fitness by increasing the allocation of carotenoids towards current reproduction. This hypothesis is not presently supported by the only two studies  in which carotenoid investment in reproduction was measured, as Møller *et al*. (2005, 2008) found reduced concentrations of yolk carotenoids in barn swallows near Chernobyl compared to control areas^34,35^. However, to the best of our knowledge, the potential effects of an accidental release of radionuclides on carotenoid allocation to a sexual signal have never been studied in any organism.

To fill this gap, we investigated carotenoid distribution in relation to radiation exposure in wild amphibians (Japanese tree frogs, *Hyla japonica*) 16 months after the Fukushima nuclear explosion. Amphibians are currently the most globally impacted group of vertebrates by human activities (especially via pollution and habitat destruction), with approximately 41% of all species threatened^35^. Their environmental sensitivity is largely due to their permeable and unprotected (by feathers, scales or hairs) skin that permits absorption of many pollutants^36^, but surprisingly the biological effects of accidental radionuclide release has never been examined in wild amphibians (only the bioaccumulation of radionuclides has been measured in this taxon^37–40^). In addition, the mechanism and function of conspicuous coloration in some amphibians, such as tree frogs, has now been explored in several studies and the carotenoid-based coloration of their vocal sacs has been shown to be a sexually selected trait^41–43^. Finally, due to their small  home range, tree frog species could be very useful for ecotoxicological studies, because individuals do not move among sites with different contamination levels. Here, we measured carotenoid concentrations in plasma, liver, and vocal sac skin samples in Japanese tree frogs that were collected from seven selected sites along a gradient of radioactivity in the Fukushima prefecture. While plasma carotenoid concentration represents a short-term snapshot of the circulating levels, liver carotenoid concentration likely reflects endogenous carotenoid reserves, since the liver (along with adipose tissue) is the major body storage site of these pigments^44^. Finally, skin carotenoid concentration represents pigments allocated to the sexual color signal in this species^43^.

Most studies on radiation exposure in wild animals have used a measurement of the ambient dose rate or activity concentrations in some components of the environment, which does not permit determination of a robust dose-response relationship for wildlife exposed to ionizing radiation in the field^45^. Therefore, we chose to estimate radiation exposure through the total dose rate (hereafter TDR for Total Individual Dose Rate) absorbed by each frog collected. This was performed to provide an appropriate description of the exposure, taking into account the contribution of all radionuclides and radiation types (alpha, beta and gamma emitters) from all exposure pathways (internal and external exposure). We then assessed: (1) how TDR varies within a site and (2) the relationship between TDR and carotenoid accumulation. Finally, since carotenoid allocation can vary with age in several taxa (three-spined sticklebacks, *Gasterosteus aculeatus*^46^; Australian painted dragons, *Ctenophorus pictus*^47^; great tits, *Parus major*^48^), we measured the age of each frog and tested for potential age-specific effects of TDR on carotenoid distribution. Given that an increased exposure to radionuclides should elevate oxidative stress^19^ and impair animal health^49^, we predicted a reallocation of carotenoids to limit these negative somatic effects of ionizing radiation. More precisely, carotenoid levels might decrease in body tissue reserves (liver) and in the sexual signal (vocal sac) but increase in circulation with an increase of TDR. Alternatively, (a) carotenoid levels may be depressed in all tissues if dietary intake and internal supplies have been depleted over time, or (b) if animals are investing carotenoids according to a terminal reproductive investment strategy, we might expect upregulation of carotenoid levels in all tissues, and especially in skin.

## Methods

### Field methods

**Figure 1:**
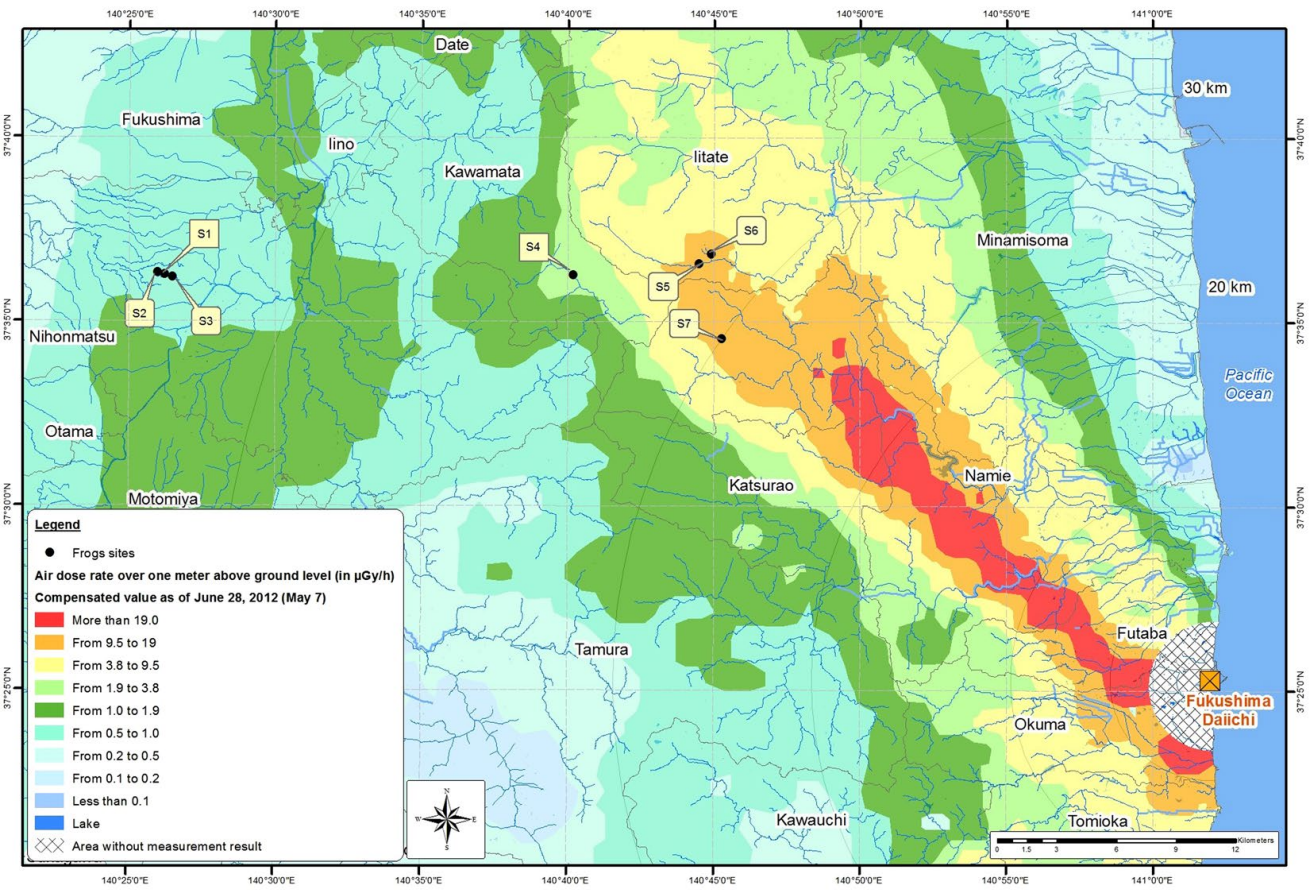
Map of ambient dose rate (µGy/h) in the Fukushima area and location of the seven sampling sites (S1–S7). The map was created by JM Métivier with ArcGis 10.3.1.

From 20 June-9 July 2012, we captured 139 male Japanese tree frogs during the breeding season at seven study sites located in the Fukushima prefecture (Fig. 1). These sites were not chosen randomly but to cover a gradient of ambient dose rates. We chose to focus our study on males since females of this species do not display ornate vocal sac coloration. Frogs were captured in flooded paddy fields surrounded by forest between 2100 and 0000 hours. After capture, individuals were kept in individual boxes (diameter: 12 cm, height: 7 cm) with a perforated cover and 2 cm of water until the next morning when they were euthanized and dissected. The ambient radiation dose rate at ground level was measured at six locations randomly selected on the bank of the paddy field at each of our seven study sites using a hand-held dosimeter (FH 40 G, Thermo Scientific). The mean (±SD) ambient radiation dose rate varied from 0.15 ± 0.04 to 7.91 ± 1.41 µGy/h (Table 1). At each of our study sites, and on the day of capture, three samples of water (i.e. 10 ml collected at a depth of approximately 2 cm) and soil (i.e., 0–5 cm layer depth on the bank of the paddy field) were collected and frozen until further analyses (see below). The three samples were then mixed to form one unique composite sample of water and soil respectively, for each study site. All procedures were performed in accordance with relevant guidelines and regulations, and approved by the Institutional Animal Care and Use Committee of the Institut de Radioprotection et de Sureté Nucléaire (IRSN; Protocol number: A1301307).

**Table 1:** Total, internal and external dose rates estimated in adult *Hyla japonica* males during the breeding season (20 June – 9 July 2012). Ambient dose rates are also indicated.

Sites	Total dose rate (µGy/h)	Internal dose rate (µGy/h)	External dose rate (µGy/h)	Ambient dose rate (µGy/h*)
S1	0.42 ± 0.22 (0.18–1.10)	0.30 ± 0.22 (0.06–0.98)	0.12 ± 0.02 (0.09–0.19)	0.61 ± 0.04
S2	0.45 ± 0.19 (0.17–0.85)	0.31 ± 0.20 (0.04–0.73)	0.14 ± 0.03 (0.12–0.20)	0.52 ± 0.09
S3	0.49 ± 0.34 (0.19–1.43)	0.33 ± 0.34 (0.03–1.27)	0.16 ± 0.02 (0.14–0.22)	0.15 ± 0.04
S4	1.13 ± 0.44 (0.59–2.13)	1.01 ± 0.44 (0.47–1.98)	0.12 ± 0.01 (0.12–0.15)	1.76 ± 0.38
S5	6.68 ± 3.87 (2.56–11.07)	6.19 ± 3.87 (2.06–10.58)	0.50 ± 0.00 (0.49–0.50)	6.49 ± 1.51
S6	10.89 ± 4.81 (4.50–22.55)	10.02 ± 4.81 (3.64–21.68)	0.87 ± 0.02 (0.85–0.93)	6.81 ± 1.52
S7	20.82 ± 7.35 (9.96–34.20)	19.22 ± 7.33 (8.38–32.62)	1.60 ± 0.04 (1.57–1.71)	7.91 ± 1.41

Mean ± SD. Range is shown in parentheses.

*Under the hypotheses of radiation coming mainly from gamma emitters, we  assumed that 1µSv/h = 1µGy/h.

### Tissue collection

To estimate body size of each frog, we measured snout-to-vent length (with a digital caliper, to the nearest 0.1 mm) and body mass (with a digital scale, to the nearest 0.1 g). Then, 100 µL of blood was collected by cardiac puncture with a heparinized capillary tube. Blood samples were centrifuged at 6000 rpm for 6 min and the plasma stored at −80 °C for later analysis. Finally, individuals were sacrificed to collect the right femur, liver and vocal sac skin. Collected tissues and the remaining carcass were then frozen and kept at −80 °C until further analyses, while the right femur was fixed in 70% ethanol.

### Skeletochronology analysis

The skeletochronological analysis followed previous procedures^45^. The tibiofibula is considered one of the best long bones for using skeletochronological techniques in hylid frogs. Muscle and skin were removed and the bone was decalcified in 4% nitric acid for 1–4 h (depending on the size of the bone), and washed in running tap water for 12 h. Cross sections of the diaphyseal region of the bone were obtained using a freezing microtome (Microtom heidelberg HM330), stained with Ehrlich’s haematoxylin, and analysed with a light microscope (Olympus CX40). Since annual periodicity in lines of arrested growth (LAGs) has been previously demonstrated^50,51^, we used these marks to estimate the age of our individuals (in years).

### Radionuclide concentrations in collected samples

Before radionuclide concentration measurements, all collected samples (i.e., water, soil and frog carcass samples) were weighed. Frog carcass samples were then dried (at 60 °C for 48 h) and were acid-wet digested in 20 mL glass vessels (liquid scintillation vials, PerkinElmer, Courtaboeuf, France) with 2 mL of HNO_3._ Vessels were placed on a sand-bath at 150 °C for 48 h; 1 mL of H_2_O_2_ was then added and the vessels were heated again at the same temperature for 24 h. This procedure was repeated until complete digestion of the samples, and digested samples were then made up to 20 mL with 2% HNO_3_. Soil samples were also dried (at 60 °C for 48 h) before their transfer into a 60 mL plastic container. Water samples were directly transferred to a 20 mL glass vessel with 2 mL of HNO_3_.

After preparation, all samples were analyzed by gamma spectrometry, equipped with a high-purity germanium detector and a multi-channel analyzer (Type P germanium crystal 170 cm^3^, Eurisys-Mesure), to measure the concentration of gamma-emitting radionuclides. The germanium detector was calibrated using several solid calibration standards of specific shape for each sample type. All samples were measured for a period varying from 10 min to 64 h. Caesium (^134^Cs and ^137^Cs) and ^110m^Ag were the only radioisotopes detectable in the samples (see more details in Supplementary Information: Table S1 and Table S2).

### Total individual dose rate estimation

We combined radionuclide activity concentrations measured in collected samples (i.e., frog carcass, water and soil samples, expressed in Bq per unit of mass) and Dose Coefficients (DCs, expressed in µGy/h per Bq per unit of mass) to estimate Total Individual Dose Rate (TDR, expressed in µGy/h) absorbed by each frog during the breeding period. The TDR reflects the energy deposited into the frog’s body per unit of time. Given that radiation sources are external (i.e. surrounding contaminated environment, such as water and soil) and internal (ingestion or dermal absorption), the intensity of this deposit is a function of radiation energy, as well as of the organisms’ shape, composition and lifestyle^52^.

DCs were calculated for both internal and external exposure using the EDEN v3 IRSN software, considering body shapes, elementary compositions of the organism (here, an individual frog) and of the environmental radiation sources (here, water and soil), for given radionuclides (here, ^134^Cs, ^137^Cs, ^110m^Ag), and according to ecologically plausible exposure scenarios. DCs allow conversion of the activity of a radionuclide in an organism or medium (Bq/mass unit) into a dose rate (Gy/time unit) and are thus specific for each radionuclide-organism combination (see more details in Supplementary Information).

The calculation of such a Total Dose Rate relies necessarily on some assumptions and simplifications. For example, DCs are estimated considering a uniform distribution of radionuclides both in media and organisms, yet this assumption is false for some radionuclides. It is well known that strontium (Sr) and iodine (I) isotopes target bones and thyroid. However, the related uncertainty has been quantified and does not exceed 30% when calculating the whole-body dose rate^53^. Regarding media, radionuclide distribution in a solid matrix may vary with depth, which is usually described by contamination profile; however, taking into account this profile for external dose assessment is not significant for ionizing radiation other than alpha^54^. The other component of the TDR calculation is the activity concentrations of radionuclides in an organism’s body and the surrounding medium. Exposure media are selected on the basis of the most ecologically plausible scenario, built from the available knowledge of the animal’s lifestyle (see supporting information). The uncertainties on the scenario are probably the main source of uncertainty. The ecological data, generally not specific to the studied site, are defined for a representative “mean” individual. The time budget allocated to this representative frog, as well as the different microhabitats under consideration, are partly arbitrary. Despite these limitations, the Total Dose Rate is acknowledged as the best compromise to assess the actual exposure of wildlife in the field to ionizing radiation^55^.

### Measurements of carotenoids

We followed prior methods for carotenoid extraction and analysis via high-performance liquid chromatography (HPLC) for our plasma and liver samples^56^. We detected four different types of carotenoids in plasma (lutein, zeaxanthin, β-cryptoxanthin and β-carotene) and five different types of carotenoids in liver (lutein, anhydrolutein, zeaxanthin, β-cryptoxanthin and β-carotene). Skin carotenoids were esterified and difficult to analyze using HPLC (due to broadly and inconsistently eluting peaks), so we used absorbance spectrophotometry (sensu Steffen and McGraw^57^) to measure total carotenoid concentration in vocal sac samples. We did not detect other red/orange/yellow pigments in initial chemical tests of skin samples, so it is reasonable to assume that vocal sac coloration is due to the presence of carotenoids (as is the case in other closely related species^41–43^). However, since we cannot exclude the possibility that vocal sac coloration might have a structural component, we recommend future studies to test for an association between skin carotenoid levels and coloration.

### Statistical analyses

#### Frog’s internal and external dose rates, and relationship between ambient dose rate and total individual dose rate estimation (TDR)

We used a Student’s paired t-test to test for a potential difference between the internal and external dose rates of individual frogs. To examine the association between external radiation levels (µGy/h) measured by a  hand-held dosimeter and estimated TDRs, we used linear mixed models (LMM) with TDR as the response variable and ambient radiation levels as the predictor. Collection site was included as a random effect.

#### Relationship between total individual dose rate estimation (TDR) and age and body condition

High radiation levels may speed up aging and decrease life expectancy, leading to populations made of younger individuals in areas with stronger radio-contamination. Similarly, body condition might be affected by the level of radiocontamination. We thus also used LMM to test whether body condition (estimated as the residuals of the log-log least-squares linear regression of body mass against snout-vent length; estimate = 1.013 ± 0.225; t = 8.509; p < 0.001) and age were associated with TDR, including TDR as the predictor variable and either body condition or age as the response variable; again collection site was included as a random factor. We used stepwise backward selection to eliminate non-significant variables (*p* > 0.05) in the final model.

#### Relationship between total individual dose rate estimation (TDR) and carotenoid contents of plasma, liver and skin

Here we used LMMs with carotenoid concentrations as response variables, TDR as the predictor, and collection site as the random effect. Frog body condition, age, and their interactions with TDR were also included as fixed effects. We used stepwise backward selection to eliminate non-significant variables. Because levels of the different carotenoid types detected in plasma were tightly positively intercorrelated within individuals, we used total carotenoid concentration for plasma in our main statistical analysis^58,59^; results for individual carotenoids are presented in ESM.

#### Total individual dose rate estimation (TDR) and correlations between carotenoid contents in plasma, liver and skin

To test whether TDR affected potential correlations between carotenoid contents in plasma, liver and skin, we built three LMMs with one of the three total carotenoid concentrations as the response variable (also including the interaction between one of the two other carotenoid concentrations and TDR) and with site as a random effect. In all analyses, TDR and the three carotenoid concentration measurements were log-transformed to meet assumptions of homoscedasticity and normality. All variables were standardized to a mean of 0 and a variance of 1. All statistical analyses were carried out using R 3.01 (R Development Core Team 2008) and the ‘lme’ procedure in the package nlme^60^ with α set at 0.05.

## Results

### Frog’s internal and external dose rates

**Figure 2:**
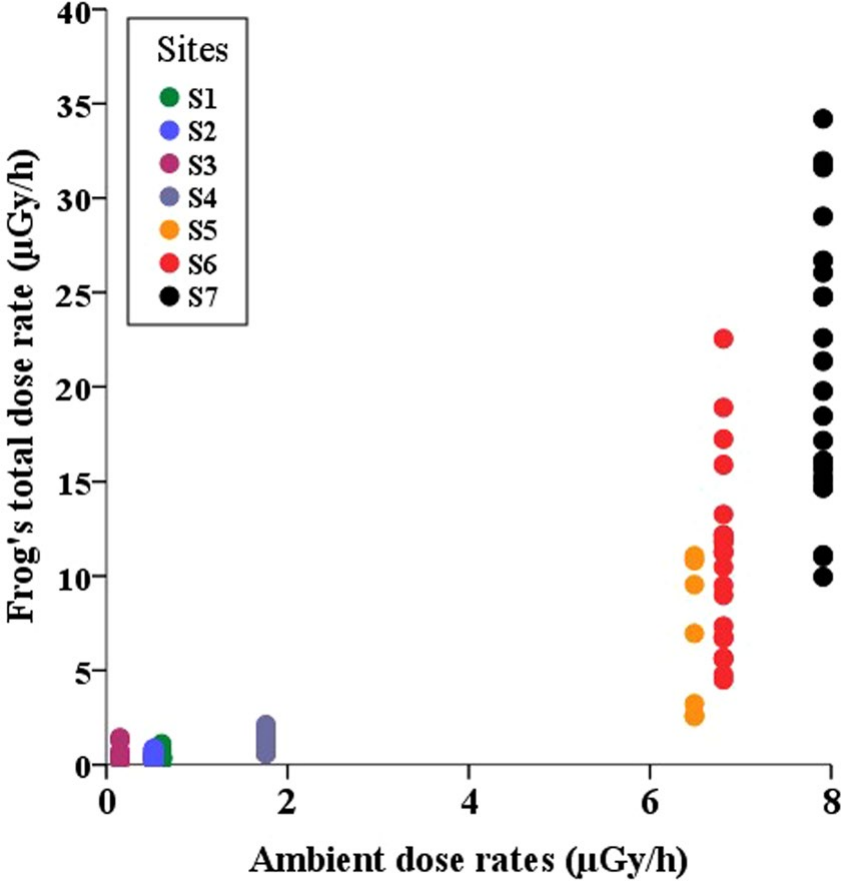
Total individual dose rate (TDR, µGy/h) in frogs and ambient dose rates at the seven sites.

The internal dose rate was significantly higher than the external dose rate (paired t-test: t = 7.7004, df = 138, p < 0.001; mean difference between internal and external = 3.67µGy/h (95% CI: [3.66; 6.20]), mean contamination for internal dose rate: 5.45 ± 8.05 µGy/h; mean contamination for external dose: 0.52 ± 0.56 µGy/h) (Table 1). In addition, TDR was characterized by a high degree of within-site variability, especially for the most radio-contaminated sites (i.e. S5, S6, and S7; Table 1 and Fig. 2). For example, the TDRs at site S6 varied from 4.50 to 22.55 µGy/h.

### Relationship between total individual dose rate estimation (TDR) and age and body condition

Body condition and age of frogs were not significantly associated with TDR (body condition: estimate = 0.332 ± 0.185, t = 1.798, df = 131, p = 0.074, age: t = −0.087 ± 0.162, df = 102, p = 0.594; see Figure S2 and Table S6 in Supplementary material). Collection site was always included as a random effect, as it always significantly correlated with the response variables (LRT tests: *p* < 0.001 in all cases).

### Total individual dose rate estimation (TDR) and total carotenoid concentration in plasma, liver and skin

TDR did not significantly correlate with carotenoid content in plasma (estimate = 0.025 ± 0.018, df = 69, t = 1.355, p = 0.180), liver (estimate = −0.006 ± 0.014, df = 93, t = −0.463, p = 0.644), or skin (estimate = −0.003 ± 0.005, df = 95, t = −0.611, p = 0.542) (see Figure S2 and Table S5 in Supplementary material). Body condition was significantly associated with skin carotenoids only, such that individuals in better body condition had more carotenoids in skin (estimate = 0.251 ± 0.096, df = 96, t = 2.619, *p* = 0.010) (Figure S2 in Supplementary material). Age was not correlated with carotenoid content in plasma (estimate = −0.182 ± 0.130, df = 62, t = −1.407, p = 0.164), liver (estimate = 0.004 ± 0.101, df = 87, t = 0.035, p = 0.972) or skin (estimate = 0.051 ± 0.046, df = 88, t = 1.106, p = 0.272).

### Total individual dose rate estimation (TDR) and correlations between carotenoid contents in plasma, liver and skin

Plasma and liver carotenoid concentrations were significantly positively correlated (estimate = 0.241 ± 0.116, df = 60, t = 2.075, p = 0.042), but carotenoid concentration in skin was not significantly correlated with either plasma (estimate = 0.388 ± 0.332, df = 61, t = 1.169, p = 0.247) or liver carotenoid concentration (estimate = 0.341 ± 0.212, df = 94, t = 1.608, p = 0.111). The interaction between TDR and skin carotenoid concentration was not significantly associated with liver carotenoid concentration (estimate = 0.226 ± 0.179, df = 92, t = 1.266, p = 0.209) or plasma carotenoid concentration (estimate = −0.130 ± 0.308, df = 59, t = −0.423, p = 0.674). The interaction between TDR and liver carotenoid concentration was not significantly associated with plasma carotenoid concentration (estimate = −0.099 ± 0.134, df = 58, t = −0.741, p = 0.462). Thus, variation in dose rate absorbed by individual frogs did not influence the correlations between carotenoid contents in the plasma, liver and skin. Collection site was always included as a random effect, as it was always significantly related to the response variables (LRT tests: *p* < 0.001 in the six cases).

## Discussion

We examined variation in carotenoid distribution among three tissues (plasma, liver and vocal sac skin) in Japanese tree frogs captured along a gradient of radioactive contamination in the Fukushima prefecture, only 16 months after the accident at the Fukushima Dai-ichi NPP. For the first time in an ecophysiological study in a radioactively contaminated natural environment, we combined the measurement of a crucial physiological process (carotenoid distribution) with an accurate estimation of the radiological dose received by each individual. We uncovered three main results: (1) high within-site variability in levels of TDR, (2) TDR levels were better explained by internal dose rate than external dose rate, (3) and no significant effects of the exposure to ionizing radiation on frog body condition or tissue carotenoid concentration, which is not comparable to findings from previous studies^21,25,35,61,62^.

### High variability in levels of total individual dose rate estimation (TDR)

A major strength of our study is the accurate quantification of an individual animal’s absorbed dose of radiation that was missing in most of the ecophysiological and evolutionary studies published after the Chernobyl and Fukushima nuclear accidents^26,34,35,63^. Bioaccumulation and subsequent internal dose rate was the main contribution to absorbed dose in our study. Omitting this contribution of the animal’s exposure, as is done when considering only ambient dose rate, can lead to an underestimation of the absorbed dose of individuals. Thus, our result highlights the importance of taking into account the absorbed dose calculation in future studies aiming to quantify dose-response relationships (even if the TDR calculations partly rely on simulations that incorporate several assumptions that can be improved over time depending on the model species). In addition, our robust dosimetry data have allowed us to show that within-site levels of individual absorbed doses are highly variable among male Japanese tree frogs, stressing the fact that only considering the ambient dose rates measured at ground level at the trapping sites may not be accurate enough to capture some of the relationships between phenotypic parameters and exposure to the artificial radionuclides released after the nuclear power plant accidents in the Ukraine and Japan. Exposure of animals to a similar ambient dose rate may in fact result in  highly different TDRs, due both to the inclusion of the internal exposure pathway and the inter-individual variability.

At least three non-exclusive hypotheses could explain high within-site variation in TDR levels. First, Japanese tree frogs use rice fields principally for breeding purposes from May to August and then move to forested areas where they hibernate (e.g. leaf litter, rodent burrows, in trees, under stones^64,65^). The rice field, forest, and hibernaculum microhabitats present different levels of contamination^4,66^, and frogs may vary in levels of individual contamination based on (1) time spent in the rice field and forest before capture and (2) the type of refuge used for hibernation. Significant variation in contamination has even been observed at the scale of the rice field^67,68^ and may impact the levels of individual contamination we measured^69^. A second potential explanation is that frog diets may vary due to inter-individual differences in foraging preferences among the wide variety of prey taxa they eat^70^ or due to differences in the spatial distribution of prey within rice fields and forests. If different types of prey contain different concentrations of artificial radionuclides, then dietary variation among frogs may generate variation in levels of their internal contamination^71^. Seasonal or size-related dietary variation (individuals consume larger prey as they grow) has been shown in other tree- and pond-frog species (i.e. *Rana nigromaculata*^72,73^ and *Hyla arborea*^74^) but our data do not show any relation between levels of individual contamination and individual body size. Thus, future studies should examine if strong inter-individual differences in foraging preferences exist in Japanese tree frogs, as has been already extensively observed in other taxa (i.e. birds and mammals^75–79^) and if the different prey items of this frog species vary in their levels of contamination. A third explanation for high intrasite variation in TDR is inter-individual differences in the bioaccumulation of radionuclides, as was shown, for example, in trout (*Oncorhynchus mykiss*) exposed to ^137^Cs under laboratory conditions^80^.

In any case, given that we found high variation in individual contamination in a species with a small  home range (most frog species have a reported maximum distance moved of less than 1 km^81^), we expect that larger, more mobile species (such as birds and mammals) should show a similar or higher variability in individual contamination at a given trapping site than what we found in Japanese tree frogs. We thus encourage future ecophysiological and evolutionary studies to use accurate measurements of individual contamination, even if it is more challenging logistically, temporally, and financially. More collaborative projects between experts in evolutionary ecology, physiology and radioecology may facilitate this methodological improvement that will help us to refine our understanding of the specific biological effects potentially associated with environmental radionuclide releases following nuclear accidents.

### No detected effects of total individual dose rate estimation (TDR) on frog traits

Contrary to expectations and to the majority of published literature that has found numerous negative effects of radio contamination on wild animals in the Chernobyl exclusion zone (mostly on birds and mammals^82,83^), we did not find significant effects of TDRs on body condition or concentrations of carotenoid pigments in body tissues of male tree frogs. We propose several hypotheses to explain this result. First, the biological consequences of the Chernobyl accident have been extensively studied but research was largely absent during the first decade following the disaster^84^. It is thus difficult to compare our results obtained from samples collected only 16 months after the Fukushima nuclear disaster with prior studies on carotenoid distribution^34,35^ or others physiological parameters^21,34,61,62,84,85^ conducted long after the Chernobyl accident. Long-term chronic exposure to radionuclides can, for example, lead to an accumulation of mutations over time^86^ or an increased population sensitivity^87^ that may not be present after short-term exposure.

Most of the studies published after the Fukushima disaster found strong effects of radioactive contamination on wild organisms (aphids, butterfly, birds, monkeys or dogs^10,13,26,63^). This work focused mostly on morphological or population abundance effects, however, such that few studies to date have assessed the consequences of these radioactive releases at the *physiological level* in wild animals. In streaked shearwaters, fledglings from a contaminated island off the coast of Fukushima displayed reduced levels of vitamin A compared to the control population, suggesting that streaked shearwaters may be more oxidatively stressed and/or suffer from antioxidant depletion^26^. However, these results should be considered cautiously since only two populations (one with a very low level of contamination: ambient dose rate of 0.143 µSv/h and one in a control, unexposed area) were compared, and thus many other confounding factors may explain a difference between these two populations. In monkeys, individuals from Fukushima had significantly lower white and red blood cell counts, hemoglobin, and hematocrit compared to monkeys from a control area (i.e. Shimokita). In this case the authors also found that the white blood cell count in immature monkeys was significantly negatively correlated with muscle radiocesium concentration (ranged from 78 to 1778 Bq/kg), suggesting an association between individual levels of contamination and hematological parameters^13^. However, Bonisoli-Alquati *et al*.^16^ showed at Fukushima approximately one year after the nuclear accident that levels of genetic damage in nestling barn swallows (*Hirundo rustica*) were not related to the activity concentrations (^134^Cs and ^137^Cs) measured in the nest material or an estimation of the nestlings’ external radiation exposure (range: 0.23–7.52 Gy/h). About 20 years after the Chernobyl nuclear accident, higher levels of genetic damage were observed in adults of the same species in contaminated sites in Chernobyl and at comparable levels of contamination between the two studies. This suggests that the potential deleterious effects of radiation exposure might be more difficult to detect in Fukushima due to, among others: (i) lower contamination levels in Fukushima than in Chernobyl and differences in the mixture and relative abundance of various radionuclides currently present in Chernobyl and Fukushima^88,89^; and (ii) differences in historical exposure (i.e. barn swallow populations studied in the Chernobyl region were exposed to radionuclides for almost 20 years longer than those studied in Fukushima). It should be noted, however, that Bonisoli-Alquati *et al*.^16^ only analyzed nestlings, which had been exposed for only a few days of life. In addition, in the present study, we only captured frogs that hatched before the nuclear accident (tree frogs start to breed when 2 years old) and it is thus possible that we did not detect any effect of exposure to radionuclides because these individuals were not exposed during their development. Indeed, embryonic development is known to be one of the most radiosensitive life stages^90^.

Amphibians have been extensively used as models to study the detrimental physiological effects of chemical pollutants^90–94^ (reviewed in Mann *et al*.^95^) or radionuclide accumulation in tissues^66,96–98^, but our study is, to the best of our knowledge, the first to use an amphibian species to monitor the physiological effects of an accidental release of artificial radionuclides in wild animals. In captivity, the evidence that amphibians are physiologically affected by radiation exposure is also scarce^99,100^. Even if amphibians should be more sensitive to pollution due to their permeable skin, we cannot exclude the possibility that they might have superior endogenous mechanisms of resistance to radioactivity than other organisms (i.e. better ability to cope with oxidative stress), but future studies should test this hypothesis before any conclusions can be drawn.

Our study is also the first to study carotenoid distribution in a non-avian or -aquatic species in an ecotoxicological context, and carotenoid levels might be less sensitive to the stress caused by pollution in amphibian species compared to other taxa. Generally, the role of carotenoids in amphibians has been understudied in the literature, but it is known that colorful red/orange/yellow skin in some amphibian species contains carotenoid pigments^101–103^ and is used to attract mates^43^. A few studies have experimentally examined the effects of dietary carotenoids on health or skin color in frogs, and these show a positive effect of carotenoid enrichment on post-metamorphic growth, skin coloration, and fecundity in red-eyed treefrogs (*Agalychnis callidryas*^104^), on larval growth in western clawed frogs (*Silurana* (*Xenopus*) *tropicalis*^105^) and on reproductive success in strawberry poison frogs (*Oophaga pumilio*^106^). Thus, though not revealed *per se* in our study here, the beneficial effects of carotenoids observed in others taxa^27^ seem to also be present in other anurans.

Tissue carotenoid distribution can be strongly affected by exposure to chemical pollution in other taxa (i.e. birds^107–110^), and Møller and colleagues^20^ even showed that barn swallows captured in Chernobyl had lower levels of carotenoids in liver, blood and egg yolk compared to birds captured in a control area (a similar result was found in great tits with concentrations of total yolk carotenoids and vitamins A and E depressed near Chernobyl compared to concentrations in a less contaminated Ukrainian study area^111^). Carotenoid distribution thus seems to be a good indicator of avian physiological stress in a polluted environment^34^. Given the aforementioned role of carotenoids on amphibian health, development and reproduction^105–107^ (see also^112^), it seems reasonable to think that carotenoid distribution is also a good indicator of environmental stress in amphibians. However, we cannot exclude the possibility that our limited understanding of the mechanisms and functions of carotenoids in tree frogs, and more generally in anuran amphibians, and a potential limited influence of carotenoid pigments on health and coloration might explain the differences between the results obtained in our study and those in previous studies of birds in Chernobyl.

More studies are needed to explore how amphibian antioxidant systems react to environmental pollution (i.e. by measuring enzymatic and non enzymatic antioxidant defenses but also oxidative damage)^113^, and especially toartificial radionuclide exposure, in order to fully understand how this stressor might affect a group of animals already threatened by habitat loss and modification^95,114,115^. In addition, a new line of research focusing on the mechanisms mediating a potential resistance to the detrimental effects associated with an exposure to artificial radionuclides seems promising if our result showing no effect of radioactive exposure in wild amphibians is confirmed by future studies in which the fitness effects of radiation exposure are measured.

In sum, here we have shown that carotenoid distribution is not related to the TDR in male Japanese tree frogs in Fukushima. To fully understand this result, but also the potential long-term fitness effects associated with this major nuclear accident, we encourage other teams to join in these investigations and expand our scope of research into the wildlife impacts of this nuclear disaster. The Fukushima prefecture has now been exposed to radioactive contamination for six years and so we also encourage research groups to continue to monitor wild populations, as already started for several taxa^10,13,16,26,63^ in order to detect the potential adaptations that wild species may develop in response to this radioactive contamination^7,116,117^ and to identify the species that may be threatened in the Fukushima prefecture because of the nuclear accident.

## Electronic supplementary material

ESM results

ESM
