# Supplementary material for: Carotenoid distribution in wild Japanese tree frogs (Hyla japonica) exposed to ionizing radiation in Fukushima
Source: Sci Rep. 2018 May 9;8:7438. doi: 10.1038/s41598-018-25495-5 (PMC5943346; doi:10.1038/s41598-018-25495-5)
Supplement: Supplementary file 1 — ESM results [file 41598_2018_25495_MOESM1_ESM.docx]

**ELECTRONIC SUPPLEMENTARY RESULTS**

CAROTENOID ALLOCATION IN WILD JAPANESE TREE FROGS (*HYLA JAPONICA*) EXPOSED TO IONIZING RADIATION IN FUKUSHIMA

Mathieu Giraudeau^1,2*^, Jean-marc Bonzom^3*^, Simon Ducatez^4^, Karine Beaugelin-seiller^3^, Pierre Deviche^1^, Thierry Lengagne^5^, Isabelle Cavalie^3^, Virginie Camilliri^3^, Christelle Adam-Guillermin^3^ and Kevin J. McGraw^1^

Supplementary table A.

Effect of TDR on concentration of each carotenoid measured in liver and plasma. We used linear mixed models with the concentration in carotenoid (log-transformed) as response variable, TDR as fixed effect, and site as random variable. We built one model per type of carotenoid.

|  | Response variable | estimate | se | df | t | p |
| --- | --- | --- | --- | --- | --- | --- |
| Plasma | Vitamin A | -0.0002 | 0.004 | 71 | -0.622 | 0.536 |
|  | Lut | 0.010 | 0.011 | 70 | 0.904 | 0.369 |
|  | Zeax | -0.0003 | 0.002 | 70 | -0.128 | 0.898 |
|  | Bcry | 0.007 | 0.005 | 70 | 1.458 | 0.149 |
|  | Bcar | 0.005 | 0.006 | 70 | 0.987 | 0.327 |
| Liver | Lut | -0.004 | 0.013 | 94 | -0.304 | 0.762 |
|  | Zeax | -0.006 | 0.010 | 94 | -0.553 | 0.582 |
|  | Ahl | -0.0003 | 0.008 | 94 | -0.032 | 0.974 |
|  | Bcry | 0.004 | 0.008 | 94 | 0.499 | 0.619 |
|  | Bcar | 0.007 | 0.010 | 94 | 0.689 | 0.493 |

Supplementary table B.

Effect of age on the concentration of each carotenoid measured in liver and plasma. We used linear mixed models with the concentration in carotenoid (log-transformed) as response variable, age as fixed effect, and site as random variable. We built one model per type of carotenoid.

|  | Response variable | estimate | se | df | t | p |
| --- | --- | --- | --- | --- | --- | --- |
| Plasma | Vitamin A | 0.002 | 0.003 | 63 | 0.852 | 0.397 |
|  | Lut | -0.067 | 0.054 | 62 | -1.242 | 0.219 |
|  | Zeax | -0.009 | 0.014 | 62 | -0.625 | 0.534 |
|  | Bcry | -0.020 | 0.028 | 62 | -0.720 | 0.474 |
|  | Bcar | -0.058 | 0.035 | 62 | -1.684 | 0.097 |
| Liver | Lut | 0.018 | 0.095 | 87 | 0.185 | 0.854 |
|  | Zeax | 0.025 | 0.075 | 87 | 0.333 | 0.740 |
|  | Ahl | -0.078 | 0.057 | 87 | -1.353 | 0.180 |
|  | Bcry | 0.002 | 0.069 | 87 | 0.033 | 0.974 |
|  | Bcar | -0.061 | 0.069 | 87 | -0.878 | 0.382 |

Supplementary table C.

Effect of body condition on the concentration of each carotenoid measured in liver and plasma. We used linear mixed models with the concentration in carotenoid (log-transformed) as response variable, body condition as fixed effect, and site as random variable. Significant effects are in bold style. We built one model per type of carotenoid.

|  | Response variable | estimate | se | df | t | p |
| --- | --- | --- | --- | --- | --- | --- |
| Plasma | Vitamin A | 0.024 | 0.017 | 71 | 1.369 | 0.175 |
|  | Lut | 0.463 | 0.398 | 70 | 1.162 | 0.249 |
|  | Zeax | -0.177 | 0.099 | 70 | -1.791 | 0.078 |
|  | Bcry | 0.083 | 0.19 | 70 | 0.436 | 0.664 |
|  | Bcar | -0.063 | 0.233 | 70 | -0.272 | 0.787 |
| Liver | Lut | 0.568 | 0.546 | 94 | 1.04 | 0.301 |
|  | Zeax | 0.477 | 0.445 | 94 | 1.072 | 0.287 |
|  | **Ahl** | **-0.615** | **0.302** | **94** | **2.034** | **0.045** |
|  | Bcry | -0.129 | 0.358 | 94 | -0.360 | 0.720 |
|  | Bcar | 0.052 | 0.399 | 94 | 0.131 | 0.896 |

Supplementary table D.

Correlations between pairs of carotenoid concentrations. Pearson correlations were tested on each pair of (log-transformed) carotenoid concentrations measured in the liver and plasma. For each test, we provide the coefficient of correlation (on top) and the p-value (underneath, in italic style). Significant correlations are in bold style.

|  |  | Plasma |  |  |  | Liver |  |  |  |  |
| --- | --- | --- | --- | --- | --- | --- | --- | --- | --- | --- |
|  |  | Lut | Zeax | Bcry | Bcar | Lut | Zeax | Ahl | Bcry | Bcar |
| Plasma | Vitamin A | -0.124 | **-0.247** | -0.215 | -0.167 | -0.115 | -0.085 | 0.085 | -0.024 | -0.124 |
|  |  | *0.280* | ***0.029*** | *0.058* | *0.144* | *0.349* | *0.485* | *0.490* | *0.848* | *0.311* |
|  | Lut | - | **0.579** | **0.472** | **0.479** | **0.296** | 0.210 | -0.055 | 0.123 | **0.300** |
|  |  |  | ***<0.001*** | ***<0.001*** | ***<0.001*** | ***0.014*** | *0.086* | *0.659* | *0.318* | ***0.013*** |
|  | Zeax | - | - | **0.587** | **0.428** | 0.229 | **0.266** | -0.069 | 0.127 | **0.289** |
|  |  |  |  | ***<0.001*** | ***<0.001*** | *0.060* | ***0.028*** | *0.577* | *0.302* | ***0.017*** |
|  | Bcry | - | - | - | **0.501** | 0.191 | 0.176 | -0.036 | **0.261** | **0.284** |
|  |  |  |  |  | ***<0.001*** | *0.119* | *0.151* | *0.769* | ***0.032*** | ***0.019*** |
|  | Bcar | - | - | - | - | 0.111 | 0.112 | 0.181 | **0.343** | **0.579** |
|  |  |  |  |  |  | *0.368* | *0.365* | *0.140* | ***0.004*** | ***<0.001*** |
| Liver | Lut | - | - | - | - | - | **0.930** | 0.145 | **0.643** | **0.555** |
|  |  |  |  |  |  |  | ***<0.001*** | *0.146* | ***<0.001*** | ***<0.001*** |
|  | Zeax | - | - | - | - | - |  | **0.214** | **0.705** | **0.595** |
|  |  |  |  |  |  |  |  | ***0.031*** | ***<0.001*** | ***<0.001*** |
|  | Ahl | - | - | - | - | - | - | - | **0.663** | **0.362** |
|  |  |  |  |  |  |  |  |  | ***<0.001*** | ***<0.001*** |
|  | Bcry | - | - | - | - | - | - | - | - | **0.731** |
|  |  |  |  |  |  |  |  |  |  | ***<0.001*** |
