# Supplementary material for: Carotenoid distribution in wild Japanese tree frogs (Hyla japonica) exposed to ionizing radiation in Fukushima
Source: Sci Rep. 2018 May 9;8:7438. doi: 10.1038/s41598-018-25495-5 (PMC5943346; doi:10.1038/s41598-018-25495-5)
Supplement: Supplementary file 2 — ESM [file 41598_2018_25495_MOESM2_ESM.doc]

**ELECTRONIC SUPPLEMENTARY MATERIAL**

Carotenoid distribution in wild Japanese tree frogs (*Hyla japonica*) exposed to ionizing radiation in Fukushima

Mathieu Giraudeau^1,2*^, Jean-Marc Bonzom^3*^, Simon Ducatez^4^, Karine Beaugelin-Seiller^3^, Pierre Deviche^1^, Thierry Lengagne^5^, Isabelle Cavalie^3^, Virginie Camilliri^3^, Christelle Adam-Guillermin^3^ and Kevin J. McGraw^1^

***Dose Coefficients (DCs) and Total individual Dose Rate (TDR) calculation***

The EDEN (Elementary Dose Evaluation for the Natural Environment) software (version 3) was used for all DC calculations. Developed and owned by IRSN, the EDEN tool was conceived to calculate dose-coefficient equivalents for non-human biota of those used for human dosimetry (Beaugelin-Seiller *et al.* 2006). On the basis of the radionuclide concentrations in biotic and abiotic components of an ecosystem, it allows one to determine the dose rate (or dose) received by any non-human organism exposed to ionizing radiation. EDEN provides DCs for any organism, any medium, for internal as well as external exposure, and for any kind of radiation (alpha, beta and gamma). The basic principle of EDEN is to determine basic mono-energy DC, corresponding to representative given energies of alpha, beta and gamma spectra, significant with regard to chronic exposure. The DC for any other energy is obtained by interpolation between the already calculated mono-energy DC. The DC corresponding to a given radiation (alpha, beta or gamma) of a given radionuclide is obtained by combining the mono-energy DCs estimated for each energy belonging to the considered spectrum. EDEN outputs compare well to results provided by similar tools and approaches (Vives I Battle *et al.* 2007; Beresford *et al.* 2008; ICRP 2008), especially for DCs related to internal exposure. More important distances exist for external exposure DC, due to diverse causes (considered energy spectra, pre-determined exposure scenarios, etc.). According to the last recommendations for effects-oriented field studies (Stark *et al.* 2017), the EDEN software allows a detailed and realistic assessment of dose rate absorbed by frogs, ensuring that this metric is appropriately determined (i.e. considering pathways and radionuclides that contribute the most to received dose).

The DC calculation requires defining exposure scenarios built on three elementary components: body shape, elementary composition (Table S3) and radionuclide composition of the organism and its environment. By convention, the shape of any organism is described by an ellipsoid defined by the length of its three axes, any other component being described by a semi-infinite layer. Any source of radiation, internal or external, is considered homogeneous in all its characteristics, e.g. its biochemical composition, its density and its radionuclide concentrations.

For each scenario component (organism and others), the elementary composition, indicating the contribution (expressed in percentage) of each chemical element to the total mass of the component, as well as its qualitative contamination (combination of radionuclides to take into consideration), has to be specified.

For the present study, DCs were determined according to the four exposure scenarios presented in Figure S1. Organisms are individual male frogs exposed during the breeding season to contaminated media that are air, water, and soil. To make easier the reading of the DC formulation ([S1] to [S3]), the term sediment was used in place of soil for scenarios where it is covered by water. These two terms were then consistently employed throughout the paper. In each case, the organism is its own internal radiation source and media were used as external radiation sources. The ellipsoid shapes of the organisms were defined from their individual sizes, directly measured on each male. We considered the three radionuclides ^134^Cs, ^137^Cs and ^110m^Ag since they were the only ones detected with germanium-γ spectrometry measurement; the radioactivity of the caesium daughter product (i.e., barium-137m) has been considered in the calculation of DCs. Exposure scenario was thus specific to each considered paddy field and DCs were specific to each male for each considered radionuclide. Due to the large number of individuals (N=139), we provide for illustration of their order of magnitude the mean values of internal and external DCs per site (Table S4). To account for the relative biological effectiveness of the different types of radiation, weighting factors were applied (10 for α-radiation, 3 for low‑β radiation (*E* < 10 keV), and 1 for other β-radiation and γ-radiation) following the suggestion from Beresford et al. (2007).

Once DCs were calculated, we converted radionuclide activity concentrations (Bq/g) into absorbed dose rates (µGy/h). We calculated internal, external and total dose rates for each individual male frog as shown below:

*TDR_tot_ = DR_int_ + DR_ext_* **[S1]**

*DR_int_ =*$\sum_{i=1}^{3} DC_{\mathrm{int}}(i).[AC(i)]$egg,i.MAsoil(i) **[S2]**

*DR_ext_ =*$\left[\begin{matrix} \sum_{i=1}^{3} OF_{water}.DC_{ext}\__{water}(i).AC_{water}(i)+OF_{soil}.DC_{ext}\__{soil}(i).AC_{soil}(i)+ \\ OF_{sed.}DC_{ext}\__{sed}(i).AC_{sed}(i)+OF_{air}.DC_{ext-shrub}(i).AC_{soil}(i) \end{matrix}\right]$ **[S3]**

*TDR_tot_* [µGy/h] total individual dose rate;

*DR_int_* [µGy/h] total internal individual dose rate;

*DR_ext_* [µGy/h] total external individual dose rate;

*i* radionuclide index (from 1 to 3: ^137^Cs, including ^137m^Ba, ^134^Cs, ^110m^Ag);

*DCint(i)* [µGy/h per Bq/g wet weight] dose conversion coefficient for internal exposure of a given male to radionuclide i;

*DC_ext-m_(i)* [µGy/h per Bq/g wet weight] dose conversion coefficient for external exposure to radionuclide i in each medium m of interest regarding the exposure scenario under consideration (m = water, soil, shrub or sediment);

*OF_m_* occupancy factor of the medium m, i.e. the fraction of time spent in a given habitat (m = water: at the water surface, m = soil: on the bank, m = sed: at the sediment/water interface, m = air: on the shrub),  such as *ΣOF_m_=1*

*AC (i)* [Bq. g^-1^ wet weight] activity concentration of radionuclide i in frog;

*AC_water_*(i) [Bq/g weight] activity concentration of radionuclide i in water;

*AC_soil_*(i) [Bq/g wet weight] activity concentration of radionuclide i in soil (and then in sediment);

**Table S1:** Activity concentrations (Bq/kg wet weight) of ^137^Cs, ^134^Cs and ^110m^Ag in frogs at each survey site.

| **Sites** |  | **^137^Cs** | **^134^Cs** | **^110m^Ag** |
| --- | --- | --- | --- | --- |
| S1 (n = 20) | Mean ± SD | 726 ± 550 | 301 ± 354 | 0 |
|  | Median | 564 | 143 | 0 |
|  | Range | 107 - 2254 | 0 - 1242 | 0 |
| S2 (n = 28) | Mean ± SD | 643 ± 413 | 230 ± 236 | 0 |
|  | Median | 526 | 248 | 0 |
|  | Range | 71 - 1697 | 0 - 826 | 0 |
| S3 (n = 19) | Mean ± SD | 622 ± 625 | 342 ± 402 | 0 |
|  | Median | 446 | 204 | 0 |
|  | Range | 53 - 2568 | 40 - 1502 | 0 |
| S4 (n = 22) | Mean ± SD | 1869 ± 824 | 1142 ± 484 | 2 ± 12 |
|  | Median | 1670 | 1061 | 0 |
|  | Range | 876 - 3631 | 525 - 2208 | 0 - 54 |
| S5 (n = 7) | Mean ± SD | 10783 ± 6696 | 7777 ± 4950 | 17 ± 45 |
|  | Median | 11288 | 8236 | 0 |
|  | Range | 3531 - 18369 | 2316 - 13329 | 0 - 118 |
| S6 (n = 21) | Mean ± SD | 17503 ± 8485 | 13053 ± 6135 | 60 ± 199 |
|  | Median | 16721 | 13678 | 0 |
|  | Range | 6455 - 38457 | 1. - 27484 | 0 - 884 |
| S7 (n = 25) | Mean ± SD | 30664 ± 12092 | 22795 ± 9049 | 11 ± 40 |
|  | Median | 28542 | 22902 | 0 |
|  | Range | 5896 - 56191 | 3892 - 42814 | 0 - 152 |

**Table S2:** Activity concentrations of ^137^Cs, ^134^Cs and ^110m^Ag in water and soil at each survey site.

| **Sites** | **Radionuclide concentrations** | | | | | |
| --- | --- | --- | --- | --- | --- | --- |
|  | Water (Bq/L) | | | Soil (Bq/kg wet weight) | | |
|  | ^137^Cs | ^134^Cs | ^110m^Ag | ^137^Cs | ^134^Cs | ^110m^Ag |
| S1 | 5.5 | ND | ND | 1630 | 1326 | ND |
| S2 | ND | ND | ND | 1497 | 1449 | ND |
| S3 | ND | ND | ND | 2154 | 1736 | ND |
| S4 | ND | ND | ND | 1635 | 1340 | ND |
| S5 | 40.5 | 28 | ND | 7600 | 5291 | ND |
| S6 | 37 | 21 | ND | 11807 | 9680 | ND |
| S7 | ND | ND | ND | 18801 | 18776 | ND |

ND: lower than detection limit (3.5 – 8 Bq/L for ^137^Cs, 4 – 6.5 Bq/L for ^134^Cs, 7.5 – 8 Bq/L for ^110m^Ag and 8 – 164 Bq/kg for ^110m^Ag).

**Table S3:** Elemental composition (% of the total mass), and density of soil, water, grass, air, and animal (adapted from Pröhl, 2003)

|  | **Soil** | **Water** | **Grass** | **Air** | **Frog** |
| --- | --- | --- | --- | --- | --- |
| Al | 5 | n.d. | n.d. | n.d. | n.d. |
| Ar | n.d. | n.d. | n.d. | 1.3 | n.d. |
| C | 1.6 | n.d. | 1.1 x 10^1^ | 1.4 x 10^-2^ | 1.9 x 10^1^ |
| Ca | 4.1 | n.d. | 5.8 x 10^-1^ | n.d. | 1.4 |
| Fe | 1.1 | n.d. | n.d. | n.d. | 1.0 x 10^-2^ |
| H | 2.1 | 1.1 x 10^1^ | 8.7 | 6.4 x 10^-2^ | 9.3 |
| K | 1.3 | n.d. | 1.7 x 10^-1^ | n.d. | 2.2 x 10^-1^ |
| Mg | n.d. | n.d. | 8.0 x 10^-2^ | n.d. | 4.0 x 10^-2^ |
| N | n.d. | n.d. | 8.2 x 10^-1^ | 7.5x10^1^ | 5.2 |
| Na | n.d. | n.d. | 3.0 x 10^-2^ | n.d. | 2.6 x 10^-1^ |
| O | 5.8 x 10^1^ | 8.9 x 10^1^ | 7.8 x 10^1^ | 2.4 x 10^1^ | 6.3 x 10^1^ |
| P | n.d. | n.d. | 7.1 x 10^-1^ | n.d. | 6.3 x 10^-1^ |
| S | n.d. | n.d. | 1.0 x 10^-1^ | n.d. | 6.4 x 10^-1^ |
| Si | 2.7 x 10^1^ | n.d. | 1.0 x 10^-2^ | n.d. | n.d. |
|  |  |  |  |  |  |
| (g/cm^3^) | 1.6 | 1 | 8.0 x 10^-3^ | 1.2 x 10^-3^ | 1.1 |

n.d.: not determined

**Table S4:** Mean (± SD) internal and external Dose Coefficients (DCs) for each site. See figure S1 for more information about the exposure scenarios used to estimate the external DCs.

| **Sites** | **Internal DC**  **(10^-11^µGy/h per Bq/wet kg)** | | | **Habitat for external DC calculation** | **External DC**  **(10^-11^µGy/h per Bq/wet kg or µGy/h per Bq/L)** | | |
| --- | --- | --- | --- | --- | --- | --- | --- |
|  | ^137^Cs | ^134^Cs | ^110m^Ag |  | ^137^Cs | ^134^Cs | ^110m^Ag |
| S1 | 877 ± 79.3 | 610 ± 4.70 | 310 ± 105 | In-water  •water  •sediment | 302 ± 6.56  150 ± 3.12 | 684 ± 7.41  404 ± 6.80 | 1165 ± 7.86  706 ± 12 |
|  |  |  |  | On-water  •water  •sediment | 220 ± 19  15.0 ± 31 | 530 ± 35  43.0 ± 83 | 950 ± 92  110 ± 170 |
|  |  |  |  | On-soil | 116 ± 38.7 | 336 ± 2.41 | 503 ± 177 |
|  |  |  |  | Above ground | 6.95 ± 0.01 | 26 ± 0.34 | 87.7 ± 1.08 |
| S2 | 906 ± 6.13 | 599 ± 71.8 | 346 ± 59.2 | In-water  •water  •sediment | 300 ± 4.75  147 ± 1.83 | 684 ± 5.25  397 ± 3.24 | 1160 ± 7.54  694 ± 5.53 |
|  |  |  |  | On-water  •water  •sediment | 230 ± 370  38.0 ± 58 | 560 ± 65  100 ± 160 | 1000 ± 84  190 ± 270 |
|  |  |  |  | On-soil | 128 ± 304 | 336 ± 2.32 | 545 ± 47.4 |
|  |  |  |  | Above ground | 8.42 ± 5.05 | 25 ± 5.09 | 87.5 ± 0.94 |
| S3 | 942 ± 62.6 | 614 ± 212 | 447 ± 214 | In-water  •water  •sediment | 276 ± 48.7  138 ± 15.8 | 649 ± 91.6  374 ± 43.4 | 1110 ± 150  658 ± 68 |
|  |  |  |  | On-water  •water  •sediment | 210 ± 22.8  16.6 ± 31.8 | 530 ± 36.6  46.4 ± 85.3 | 953 ± 56  88.5 ± 147 |
|  |  |  |  | On-soil | 124 ± 2.65 | 331 ± 4.07 | 581 ± 34.1 |
|  |  |  |  | Above ground | 7.47 ± 1.26 | 27.3 ± 3.07 | 89.1 ± 3.95 |
| S4 | 930 ± 49.2 | 598 ± 179 | 408 ± 167 | In-water  •water  •sediment | 285 ± 38.2  143 ± 9.49 | 666 ± 71.7  387 ± 25.6 | 1140 ± 117  677 ± 40.9 |
|  |  |  |  | On-water  •water  •sediment | 207 ± 6.05  17.7 ± 41.2 | 522 ± 3.32  25.8 ± 3.98 | 906 ± 198  52.9 ± 6.56 |
|  |  |  |  | On-soil | 125 ± 2.62 | 333 ± 4.76 | 590 ± 30.2 |
|  |  |  |  | Above ground | 7.29 ± 1.10 | 26.8 ± 2.39 | 87.5 ± 2.57 |
| S5 | 924 ± 3.07 | 628 ± 3.08 | 366 ± 3.92 | In-water  •water  •sediment | 296 ± 0.834  143 ± 1.43 | 696 ± 3.72  388 ± 3.60 | 1180 ± 7.12  678 ± 7.81 |
|  |  |  |  | On-water  •water  •sediment | 203 ± 1.66  8.39 ± 0.074 | 519 ± 1.73  24.3 ± 0.22 | 955 ± 2.23  50.7 ± 0.37 |
|  |  |  |  | On-soil | 122 ± 0.63 | 331 ± 0.76 | 598 ± 2.02 |
|  |  |  |  | Above ground | 6.86 ± 0.10 | 25.7 ± 0.30 | 87.2 ± 1.53 |
| S6 | 924 ± 35.9 | 600 ± 142 | 384 ± 122 | In-water  •water  •sediment | 290 ± 28.1  144 ± 2.30 | 675 ± 53  393 ± 4.78 | 1150 ± 85.1  687 ± 8.85 |
|  |  |  |  | On-water  •water  •sediment | 207 ± 4.59  8.68 ± 1.08 | 522 ± 2.73  2.51 ± 2.84 | 936 ± 76.5  74.3 ± 103 |
|  |  |  |  | On-soil | 124 ± 2.59 | 333 ± 4.39 | 595 ± 19.7 |
|  |  |  |  | Above ground | 7.06 ± 0.92 | 26.2 ± 1.81 | 87.7 ± 1.79 |
| S7 | 940 ± 61 | 672 ± 125 | 442 ± 207 | In-water  •water  •sediment | 276 ± 47  143 ± 8.71 | 646 ± 87.2  389 ± 23.5 | 1110 ± 142  681 ± 37.4 |
|  |  |  |  | On-water  •water  •sediment | 207 ± 5.96  9.24 ± 1.84 | 523 ± 3.15  26.6 ± 4.85 | 945 ± 21.8  54.2 ± 7.82 |
|  |  |  |  | On-soil | 125 ± 2.10 | 334 ± 6.31 | 594 ± 22.1 |
|  |  |  |  | Above ground | 7.48 ± 1.48 | 27.0 ± 3.04 | 87.9 ± 2.70 |

**Table S5:** Average and standard deviation of total carotenoid concentrations at each site (sample sizes of frogs per site are given in parentheses).

| **Sites** | **Total vocal sac carotenoid concentration (µg/g)** | **Total plasma carotenoid concentration (µg/mL)** | **Total liver carotenoid concentration (µg/g)** |
| --- | --- | --- | --- |
| S1 | 27.799 ± 1.352 (n = 18) | 4.950 ± 0.843 (n = 9) | 22.678 ± 3.666 (n = 18) |
| S2 | 27.886 ± 2.748 (n = 23) | 4.484 ± 0.803 (n = 16) | 28.746 ± 2.499 (n = 22) |
| S3 | 43.694 ± 5.319 (n = 5) | 2.497 ± 0.499 (n = 13) | 34.964 ± 8.030 (n = 5) |
| S4 | 36.551 ± 3.968 (n = 16) | 2.049 ± 0.381 (n = 14) | 34.036 ± 5.995 (n = 16) |
| S5 | 41.573 ± 5.930 (n = 7) | 1.560 ± 0.730 (n = 2) | 16.570 ± 2.284 (n = 7) |
| S6 | 33.231 ± 2.811 (n = 15) | 1.595 ± 0.372 (n = 6) | 19.641 ± 4.930 (n = 15) |
| S7 | 30.617 ± 3.220 (n = 20) | 4.976 ± 0.493 (n = 18) | 29.404 ± 3.643 (n = 19) |

**Table S6:** Standard deviation of the random effect site (Site sd) and residual standard deviation (Residual sd) for models including either TDR or carotenoid concentrations as the response variables. Note that all response variables were log-transformed.

| **Response variable** | **Fixed effect included in the model** | **Site sd** | **Residual sd** |
| --- | --- | --- | --- |
| TDR | Body condition | 1.536 | 0.599 |
| TDR | Age | 1.538 | 0.458 |
| TDR | - | 1.681 | 0.455 |
| Vocal sac carotenoid concentration | Body condition | 9.940 * 10^-6^ | 0.390 |
| Liver carotenoid concentration | - | 0.400 | 1.073 |
| Plasma carotenoid concentration | - | 0.201 | 0.848 |

**Figure S1:** Exposure scenarios used to estimate the Dose Coefficients (DCs) applied to each male frog during the breeding period. Values in parentheses indicate the depth of the different microhabitats, OF (occupancy factor) indicates the ratio of time (based on field observations) that frogs spent in each habitat: (A) at the soil-water interface; (B) at the water surface; (C) on the ground and (D) on grass at 50 cm above ground.


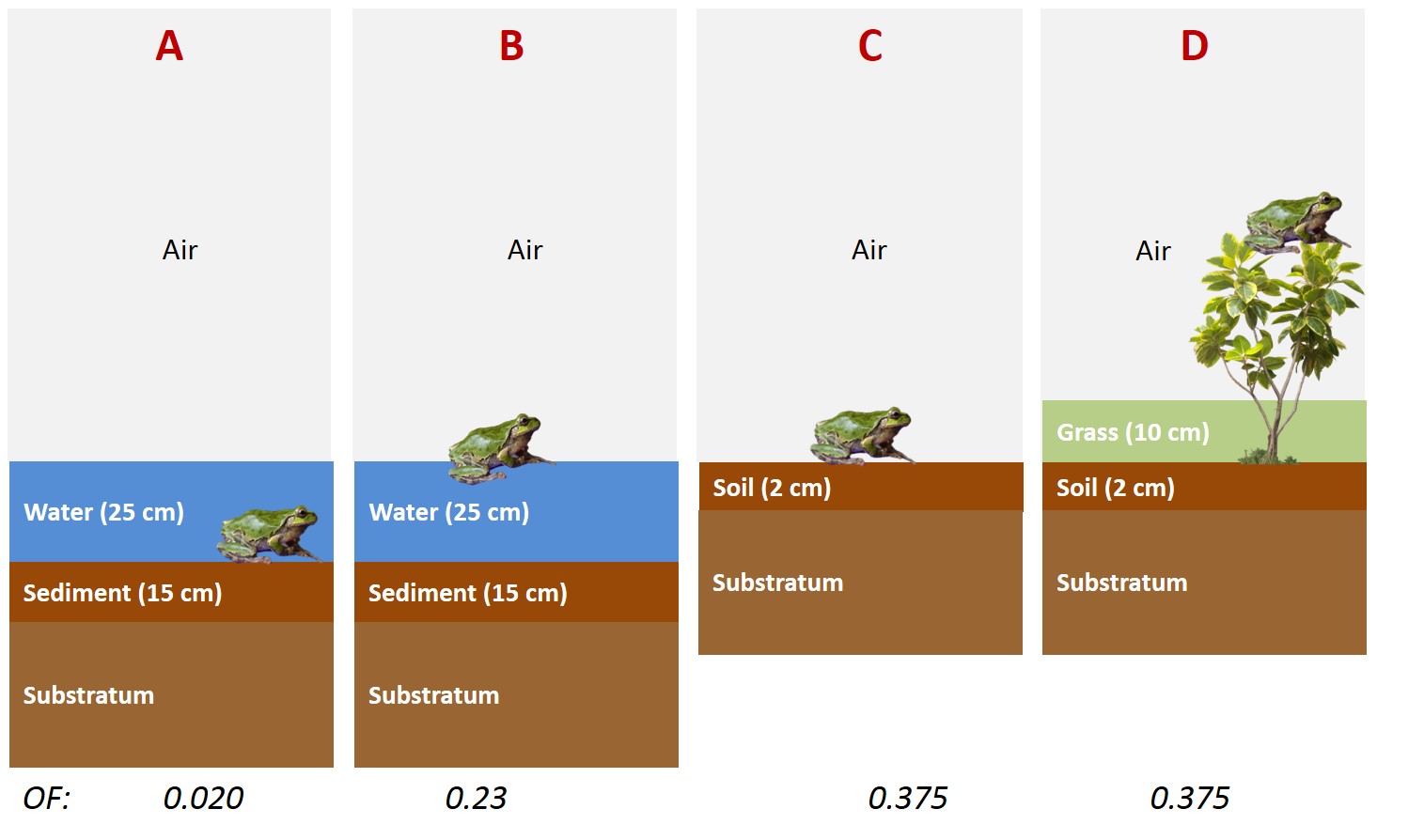


**Figures S2**: Relationships between total individual dose rate (TDR, µGy/h) in frogs collected at seven sites and age (A), body condition (B), and total concentrations of carotenoids in plasma (C), liver (D), and vocal sac (E).


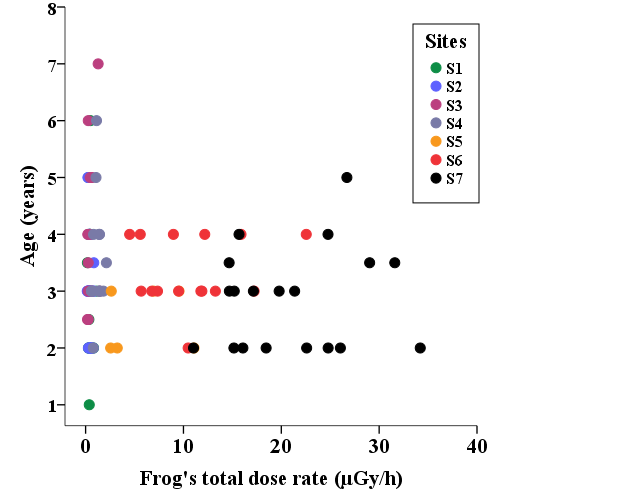


(A)


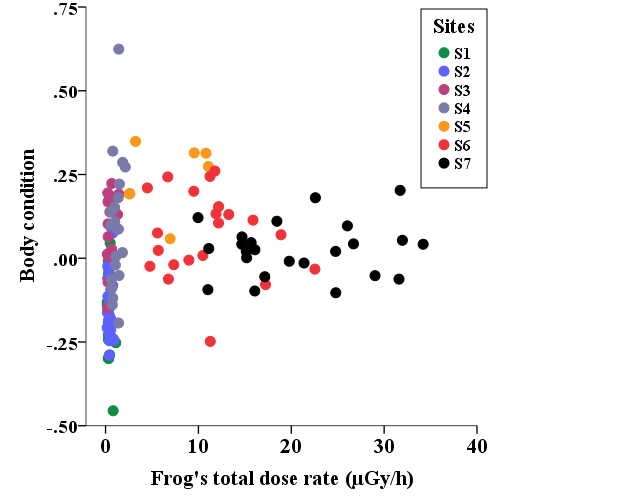
(B)


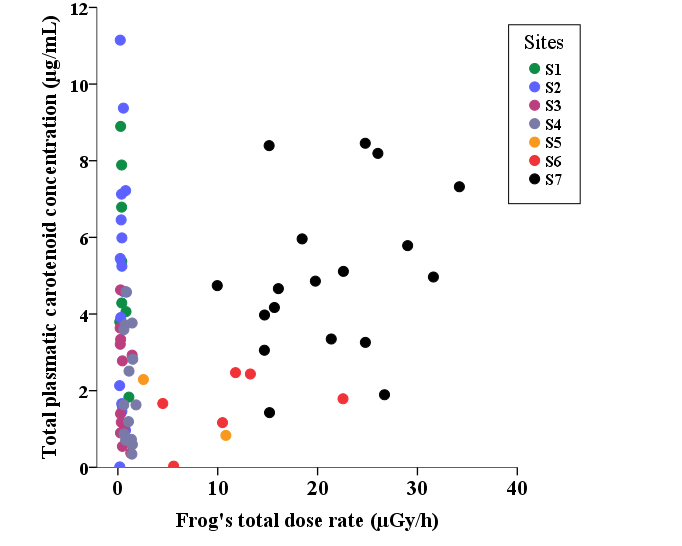
(C)


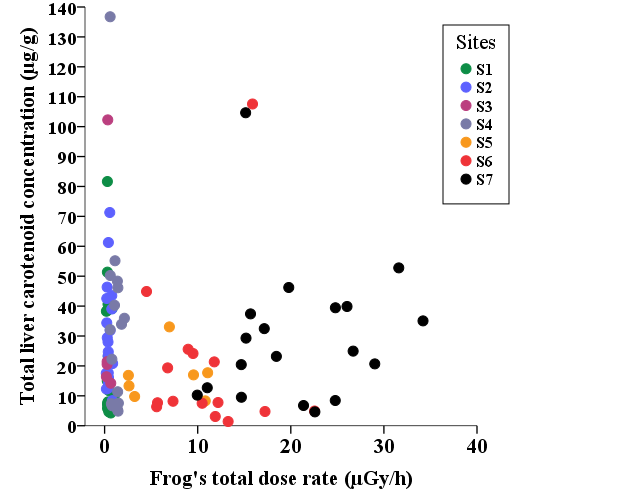
(D)


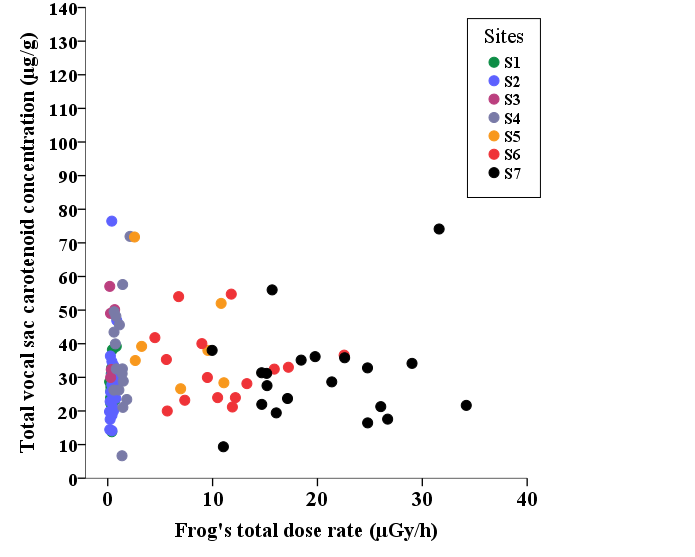
(E)

**References**

Beaugelin-Seiller, K., Jasserand, F., Garnier-Laplace, J., Gariel, J.-C. Modelling radiological dose in non-human species: principles, computerization and application. *Health Phys.*, **90**(5), 484-493 (2006).

Beresford, N.A., Brown, J., Copplestone, D., Garnier-Laplace, J., Howard, B., Larsson, C.-M., Oughton, D., Pröhl, G., Zinger, I. (Eds.). D-ERICA: An Integrated Approach to the assessment and management of environmental risks from ionising radiation. Description of purpose, methodology and application. EC project contract no. FI6R-CT-2004-508847 (2007) <https://wiki.ceh.ac.uk/download/attachments/115017395/D-Erica.pdf?version=1>

Beresford, N.A., Balonov, M., Beaugelin-Seiller, K., Brown, J., Copplestone, D., Hingston, J.L., Horyna, J., Hosseini, A., Howard, B.J., Kamboj, S., Nedveckaite, T., Olyslaegers, G., Sazykina, T., Vives i Batlle, J., Yankovich, T.L., Yu. C. An international comparison of models and approaches for the estimation of the radiological exposure of non-human biota. *Appl. Radiat. Isot.*, **66**, 1745-1749 (2008).

ICRP. Environmental protection: the concept and use of reference animals and plants. ICRP Publication 108. *Ann. ICRP* **38** (4-6) (2008).

Stark, K., Goméz-Ros, JM., Vives I Batlle, J., Lindbo Hansen, E., Beaugelin-Seiller, K., Kapustka, L.A., Wood, M.D., Bradshaw, C., Real, A., McGuire, C., Hinton, T.G. Dose assessment in environmental radiological protection: State of the art and perspectives. *J. Environ. Radioact.* **175-176, 105-114** (2017).

Vives i Batlle, J., Balonov, N., Beaugelin-Seiller, K., Beresford, N. A., Brown, J., Cheng, J.-J., Copplestone, D., Doi, M., Filistovic, V., Golikov, V., Horyna, J., Hosseini, A., Howard, B. J., Jones, S. R., Kamboj, S., Kryshev, A, Nedveckaite, T., Olyslaegers, G., Pröhl, G., Sazykina, T., Ulanovsky, A., Vives Lynch, S., Yankovich, T., Yu, C. Inter-comparison of absorbed dose rates for non-human biota. *Radiat. Environ. Biophys.*, **46**(4), 349-373 (2007).

Pröhl G, eds. Dosimetric models and data for assessing radiation exposures to biota. Delivrable 3. FASSET – project within the EC 5^th^ Framework Programme, contract No FIGE-CT-2000-00102. 103 p. (2003).
